# Copolymer-Green-Synthesized Copper Oxide Nanoparticles Enhance Folate-Targeting in Cervical Cancer Cells In Vitro

**DOI:** 10.3390/polym15102393

**Published:** 2023-05-20

**Authors:** Keelan Jagaran, Moganavelli Singh

**Affiliations:** Nano-Gene and Drug Delivery Laboratory, Discipline of Biochemistry, University of KwaZulu-Natal, Private Bag X54001, Durban 4000, South Africa; 215055447@stu.ukzn.ac.za

**Keywords:** cervical cancer, copper oxide, green synthesis, cytotoxicity, gene delivery, folate targeting

## Abstract

Cervical cancer is fast becoming a global health crisis, accounting for most female deaths in low- and middle-income countries. It is the fourth most frequent cancer affecting women, and due to its complexity, conventional treatment options are limited. Nanomedicine has found a niche in gene therapy, with inorganic nanoparticles becoming attractive tools for gene delivery strategies. Of the many metallic nanoparticles (NPs) available, copper oxide NPs (CuONPs) have been the least investigated in gene delivery. In this study, CuONPs were biologically synthesized using *Melia azedarach* leaf extract, functionalized with chitosan and polyethylene glycol (PEG), and conjugated to the targeting ligand folate. A peak at 568 nm from UV-visible spectroscopy and the characteristic bands for the functional groups using Fourier-transform infrared (FTIR) spectroscopy confirmed the successful synthesis and modification of the CuONPs. Spherical NPs within the nanometer range were evident from transmission electron microscopy (TEM) and nanoparticle tracking analysis (NTA). The NPs portrayed exceptional binding and protection of the reporter gene, pCMV-*Luc*-DNA. In vitro cytotoxicity studies revealed cell viability >70% in human embryonic kidney (HEK293), breast adenocarcinoma (MCF-7), and cervical cancer (HeLa) cells, with significant transgene expression, obtained using the luciferase reporter gene assay. Overall, these NPs showed favorable properties and efficient gene delivery, suggesting their potential role in gene therapy.

## 1. Introduction

Cancer therapy is an ongoing challenge to mitigate the complexities associated with this dreaded disease, which is the second leading cause of death globally. Cancer accounts for an alarming 18.1 million new cases, with 9.6 million deaths annually. Cervical cancer poses a threat to women worldwide, being the fourth most frequent cancer affecting women, with 311,000 deaths worldwide and an estimated 570,000 cases in 2018 [1].

The human papillomavirus (HPV), a compromised immune system, and smoking are a few mentioned determinant conditions closely related to this disease [2,3]. Treatment options for cervical cancer depend on the cancer stage, the tumor’s size and location, and the patient’s health. Standard treatment options include radiation therapy, surgery, and chemotherapy, which increase the financial distress experienced by cancer patients, above and beyond the side effects caused. Hence, women in low- and middle-income countries portray a significantly higher mortality rate than those in high-income countries [3,4]. It is thus imperative to create innovative approaches to reduce this alarming statistic. Reducing the invasiveness of the current diagnostic and treatment procedures is urgently required.

The amalgamation of medicine and nanotechnology has become a highly efficient alternative to traditional chemotherapy. In addition, targeted gene/drug therapy and immunotherapy have attracted much attention. This has necessitated the formulation of safe and effective delivery systems. Nanoparticles (NPs) have gained popularity and have been investigated for the delivery and release of pharmacologically active agents such as genes or drugs [5]. Importantly, metal NPs have undergone the most extensive studies, with gold NPs being the most employed owing to their favorable properties [6]. Copper (Cu)-based NPs have not been thoroughly investigated as therapeutic carriers. Cu, a trace element and essential nutrient, is a good conductor of heat and electricity and possesses favorable antibacterial properties. Besides its use as a metabolic cofactor, Cu has been reported to be a metalloallosteric regulator and involved in signaling pathways [7]. Cu has been reported to have low toxicity, good biocompatibility, be readily available at lower costs, and be oxidation-resistant, making it an attractive prospect for delivering therapeutic drugs and genes [8,9]. Hence, exploiting Cu on the nanoscale may benefit therapeutic use. Furthermore, Cu-based NPs are relatively stable when bound to polymers, making them suitable for use in nanomedicine [10].

Compared to other noble metals, copper tends to become easily oxidized in the presence of air. Copper-based NPs portray unique properties in the nanoscale range. These NPs contain a single-phase tenorite with a narrow band gap of 1.7 eV [11]. They are thus used in various medical applications owing to their antiviral, antibacterial, and possibly anticancer properties [12,13,14,15]. CuONPs also have the potential to be used as MRI-ultrasound dual imaging contrast agents. However, CuONPs have not been extensively studied in cancer gene therapy compared to the other metallic NPs. Hence, this study was undertaken to assess their potential in gene delivery.

The study of green chemistry has significantly influenced the synthesis of metallic and inorganic NPs. NPs can be synthesized both chemically and biologically, with the biological method portrayed as being the least toxic approach. Various plant compounds or extracts have been used since they contain many secondary metabolites that enable them to reduce metallic ions and heavy metals. The leaves, stems, bark, fruit, and seeds have been more commonly used in NP synthesis [16]. Biological syntheses have been found to produce NPs rapidly with increased stability and varied shapes and sizes [17]. It has been reported that the reducing properties of plant material in NP synthesis depend on the antioxidant content of the specific part of the plant [16]. The green synthesis of metals or metal oxide NPs is advantageous due to the lack of toxic reducing agents such as sodium borohydride and hydrazine, which are also hazardous to the environment [18]. Due to the increased interest in waste minimization or elimination via implemented fundamental properties of green chemistry, the biological synthesis of NPs is a desirable approach.

In this study, the green synthesis approach uses the extract of the leaves of *Melia azedarach* (*M. azedarach*), or syringa, as it is commonly known. Syringa has been previously identified to contain polyphenolic compounds portraying remarkable antioxidant activities common to the Meliaceae family. This plant species has antimalarial, antifungal, antiviral, antibacterial, antifeedant, antifertility, and anticancer properties [19]. The extract of the syringa leaves has shown strong antiproliferative properties against cancer cells, both in vitro and in vivo, via the induction of tumor necrosis factor-α (TNF-α) and the light-chain 3(LC3)-11 autophagosomal proteins. This has been shown to suppress tumor growth and induce autophagy [20]. *M. azedarach* displays favorable biological activities, is readily available, and has excellent reduction capabilities. It has antioxidant, antimalarial, antihepatotoxic, antibacterial, antiparasitic, and antiulcer properties [21]. This is anticipated to enrich the CuONPs and produce a synergistic therapeutic effect. Owing to *M. azedarach*’s good biological properties, there has been substantive progress in its use in anticancer studies, warranting further studies, especially in gene therapy.

The success of gene therapy relies on the ability of NPs to compact, protect, and safely deliver the therapeutic gene to the desired target cell. The present study acquired this through surface modifications via the conjugation of cationic polymers. The advantages of using chitosan (Cs) as a functionalizing polymer for NPs include its good biodegradability, biocompatibility, minimal toxicity, and the presence of -NH2 and -OH groups in its backbone for the conjugation of biomolecules [22,23]. Folic acid, or folate (F), can be conjugated to NPs to enable receptor-mediated endocytosis via folate receptors (FRs) which are overexpressed in many human cancers, including those of the kidney, cervix, brain, breast, and lungs [24,25]. Hence, the folate-receptor-targeting of HeLa cells can achieve enhanced cellular uptake and transfection. Modification with polyethylene glycol (PEG) improves steric stabilization and prolongs systemic circulation with decreased immunogenicity [26,27].

Although some progress has been made using NPs in gene therapy, the Food and Drug Administration (FDA) has put forth several challenges. These include NP aggregation in physiological fluids, cellular internalization, biocompatibility, biodegradation, and endosomal escape [28]. Hence, further research investigating various NPs is imperative. The present study is a proof-of-concept study aimed at determining the efficiency of biologically synthesized folate–chitosan–copper oxide NPs (F-Cs-CuONPs) and PEG-F-Cs-CuONPs in the delivery of a reporter gene (pCMV-*Luc*-DNA) to cervical cancer (HeLa) cells. Importantly, no studies to date have used such green-synthesized CuONPs for targeted gene delivery to cervical cancer cells in vitro.

## 2. Materials and Methods

### 2.1. Materials

Copper nitrate (Cu(NO_3_)_2_, Mw: 187.56 g·mol^−1^), sodium sulfide (Na_2_S, Mw: 78.05 g·mol^−1^), dimethyl sulfoxide (DMSO), 3-[4,5-dimethylthiazol-2-yl]-2.5-diphenyltetrazolium bromide (MTT), phosphate-buffered saline tablets (PBS, (140 mM NaCl, 10 mM phosphate buffer, 3 mM KCl)), PEG2000, ethidium bromide, bromophenol blue, xylene cyanol, sodium dodecyl sulfide (SDS), 1-ethyl-3-(3-dimethyl aminopropyl) carbodiimide (EDC), and *N*-hydroxysuccinimide (NHS) were sourced from Merck (Darmstadt, Germany). Chitosan (>75% DD, Mw: 25 kDa) and folic acid were purchased from Sigma-Aldrich (St. Louis, MO, USA), and the luciferase assay kit was supplied by Promega Corporation (Madison, WI, USA). Eagle’s minimum essential medium (EMEM), antibiotic mixture (penicillin (10,000 U/mL)), streptomycin (10,000 µg/mL), amphotericin (25 µg/mL), and trypsin-versene-EDTA were obtained from Lonza BioWhittaker (Walkersville, MD, USA). Sterile fetal bovine serum (FBS) was purchased from GIBCO, Life Technologies LTD, Inchinnan, UK. Plasmid pCMV-*Luc* DNA (6.2 kbp) was obtained from Plasmid Factory (Bielefeld, Germany). Human breast adenocarcinoma (MCF-7), cervical carcinoma (HeLa), and embryonic kidney (HEK293) cells were originally purchased directly from the American Type Culture Collection (ATCC, Manassas, VA, USA). Ultrapure (18 Mohm) water (Milli-Q50, Millipore, France) was utilized in all assays, and all general reagents were of analytical grade.

### 2.2. Sample Collection and Preparation

#### 2.2.1. Sample Collection

**Figure 1:**
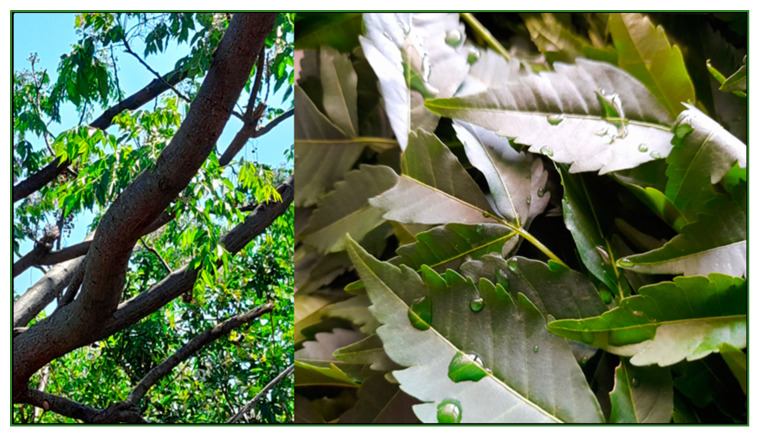
Photograph of *Melia azedarach*.

The *M. azedarach* (syringa) leaves were collected in January 2020 from the Durban area, KwaZulu-Natal, South Africa, coordinates: 29°50′48.195″ S; 31°0′34.233″ E. Young leaves were obtained from the tree (Figure 1) and placed in a sealed plastic bag. This allowed for the inhibition of transpiration and therefore the drying out of the leaves in transit to the laboratory. A *M. azedarach* L. (Meliaceae) specimen was deposited in the Ward Herbarium (voucher: K. Jagaran Collection Number 1), Westville Campus, UKZN. The species was identified and confirmed by the curator of the Ward Herbarium, School of Life Sciences, UKZN.

#### 2.2.2. Preparation of *M. azedarach* Leaf Extract

The leaves of the *M. azedarach* plant were prepared as previously described [29]. The leaves were thoroughly washed with distilled water soon after collection and blotted dry using a paper towel. Approximately 10 g of the leaves were cut into small pieces, transferred to a beaker containing 40 mL of 18 Mohm water, and heated to between 80 and 90 °C, with constant stirring for 15 min, until a green-colored liquid solution was noted. The leaf extract was filtered (Whatmann Filter paper no. 5) and stored in a freezer at −4 °C until further use.

### 2.3. Synthesis and Chitosan (Cs) Functionalization of Copper Oxide Nanoparticles (CuONPs)

**Figure 2:**
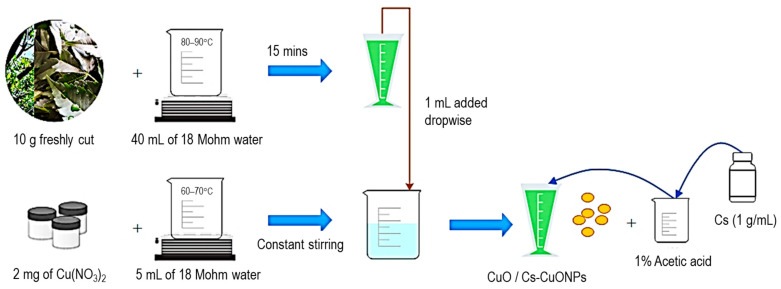
Schematic representation of the synthesis of CuO and Cs-CuONPs.

A copper nitrate (Cu (NO_3_)_2_) solution was prepared by dissolving 2 mg of Cu(NO_3_)_2_ in 5 mL of 18 Mohm water. The solution was heated to 60–70 °C with constant stirring, followed by the dropwise addition of 1 mL of the leaf extract until a light green color was visible. This indicated the formation of the CuONPs [30]. For chitosan (Cs) functionalization, Cs solution (1 g/mL in 1% acetic acid) was added to the nanoparticles with stirring at predetermined ratios of 1:1, 1:2.5, and 1:3.5 (*v*/*v*) for optimal binding [31]. A scheme for the above synthesis is illustrated in Figure 2.

### 2.4. Formation of Folate–Chitosan–Copper Oxide (F-Cs-CuONPs) and PEG-F–Chitosan–Copper Oxide Nanoparticles (PEG-F-Cs-CuONPs)

To a solution of folic acid (10 mg/500 µL in DMSO), 15 mg of 1-ethyl-3-(3-dimethylamino propyl) carbodiimide (EDC) (0.19 g/mL), 12 mg of *N*-hydroxysuccinimide (NHS) (0.26 g/mL), and 5 mL of 18 Mohm water were added. The solution was covered with foil and stirred overnight at room temperature. To 2 mL of the previously prepared Cs solution, 0.5 mL of the folic acid solution was added. The CuONPs were added dropwise to the folate–chitosan (F-Cs) solution in a ratio of 1:3.5, forming the F-Cs-CuONPs [32].

Subsequently, PEGylation of the F-Cs-CuONPs was achieved by adding 2% Polyethylene glycol dropwise, followed by 2 h of stirring for successful conjugation. To remove unreacted products, dialysis (MWCO 12,000 -14,000 Da) was performed against 18 Mohm water [33].

### 2.5. Characterization of Nanoparticles and Nanocomplexes

#### 2.5.1. UV-Vis Spectroscopy and X-ray Diffraction (XRD)

The absorption spectra of the CuO, Cs-CuO, F-Cs-CuO, PEG-Cs-CuO, and PEG-F-Cs-CuONPs were recorded using a UV-vis spectrophotometer (Jasco V-730, JASCO Corporation, Hachioji, Japan) over a wavelength range of 200–800 nm. The results were confirmed by those reported in the literature. X-ray diffraction (XRD) was conducted using a Malvern Panalytical Aeris diffractometer (Malvern, Worcestershire, UK).

#### 2.5.2. Fourier-Transform Infrared Spectroscopy (FTIR)

FTIR spectroscopy of all NPs was performed using a PerkinElmer Spectrum 100 FTIR spectrophotometer (Shelton, CT, USA), fitted with a universal attenuated total reflection (ATR) sampling accessory. A single droplet of each colloidal nanoparticle (NP) was placed in the instrument stage, and the spectra were analyzed from 4000–400 cm^−1^ using 64 co-added scans at a resolution of 4 cm^−1^.

#### 2.5.3. Transmission Electron Microscopy (TEM)

TEM was used to determine the ultrastructural morphology of the CuO and its functionalized counterparts. Images were captured using a JEOL-JEM T1010 electron microscope and analyzed using iTEM Soft Imaging Systems (SIS) Mega view III (JEOL, Tokyo, Japan). One drop of the respective sample was placed onto a carbon-coated copper grid and air-dried at room temperature. After that, images were viewed, captured, and analyzed.

#### 2.5.4. Nanoparticle Tracking Analysis (NTA)

Particle size and zeta (ζ) potential were assessed using NTA [34], operating at 24 V at 25 °C. A 1:100 dilution of the NPs in 18 Mohm water was used and evaluated using a Nanosight NS500 complete with NTA version 3.0 software (Malvern Instruments, Worcestershire, UK). The software utilized the Stokes–Einstein equation and Smoluchowski approximations to provide the particle size distribution and zeta potentials, respectively.

### 2.6. Nucleic Acid Binding Studies

#### 2.6.1. Electrophoretic Mobility Shift Assay

Nanocomplex formation (Cs-CuO/pDNA, F-Cs-CuO/pDNA, PEG-Cs-CuO/pDNA, and PEG-F-Cs-CuO/pDNA) was assessed using the mobility shift electrophoresis assay [35]. The concentration of pCMV-*Luc* DNA (pDNA, 0.25 µg/µL) remained constant, while the amount of the NPs was gradually increased. The nanocomplexes were incubated for 1 h at 25 °C to ensure effective binding of the NP to the pDNA. This was followed by electrophoresis at 50 V for 90 min on a 1% agarose gel pre-stained with ethidium bromide (1 µg/mL) using BioRad mini-sub electrophoresis apparatus (BioRad, Richmond, VA, USA). Agarose gels were viewed, and images were captured using a Vacutec Syngene G: Box Bioimaging System (Syngene, Cambridge, UK). Three NP/pDNA binding ratios obtained were subsequently used, viz., sub-optimum, optimum, and supra-optimum binding ratios.

#### 2.6.2. Nuclease Protection Assay

The serum nuclease protection assay used fetal bovine serum (FBS) to investigate the ability of the NPs to protect the bound pDNA from enzyme digestion [35]. The sub-optimum, optimum, and supra-optimum binding ratios (Section 2.6.1) were used. FBS to a final concentration of 10% was then added. A positive control (naked pDNA) and a negative control (FBS-treated pDNA) were included in the assay. The samples were incubated at 37 °C for 4 h, followed by adding EDTA (10 mM) to halt the enzyme reaction. After that, 0.5% SDS was introduced, and the reaction was allowed to proceed for 20 min at 55 °C to facilitate the release of the DNA. Following the incubation, agarose gel electrophoresis was conducted as previously described (Section 2.6.1).

#### 2.6.3. Ethidium Bromide Intercalation Assay

This assay evaluated [35] the degree of complexation of the pDNA by the NPs. The relative fluorescence was measured using a Glomax^®^ multi-detection system (Promega Biosystems, Sunnyvale, CA, USA). Approximately 2 µL of ethidium bromide was added to 100 µL of HBS in a well of a black 96-well plate. The fluorescence was measured at an excitation wavelength of 520 nm and an emission wavelength of 600 nm. This established a baseline fluorescence of 0%. Thereafter, 1.2 µg (4.8 µL) of pDNA was added, and the measured fluorescence was set as 100%. The NPs (1 µL at a time) were added, and the fluorescence measurements were recorded until a plateau in fluorescence was reached.

### 2.7. MTT Cell Viability Assay

The MTT cell viability assay was based on that originally described [36]. HeLa, HEK293, and MCF-7 cells at/near confluency were trypsinized and seeded into 48-well plates at a density of 2.5 × 10^5^ cells/well. Cells were incubated at 37 °C for 24 h in 5% CO_2_. Nanocomplexes at the sub-optimum, optimum, and supra-optimum (*w*/*w*) ratios obtained from the electrophoretic mobility shift assay were added to their respective wells in triplicate and incubated at 37 °C for 48 h. A positive control containing untreated cells was included. After incubation, the medium in each well was replaced with 200 µL fresh growth medium containing 20 µL MTT reagent (12 mM in PBS), and cells were incubated at 37 °C for 4 h. The growth medium infused with MTT was then removed, followed by the solubilization of the formazan crystals using 200 µL DMSO. Absorbance at 570 nm was then measured (Mindray MR-96A, Vacutec, Hamburg, Germany), and cell viability was calculated as follows:

$$\%\;\mathrm{Cell}\;\mathrm{Viability}=\frac{\left[A570nm\;treated\;cells\right]}{\left[A570nm\;untreated\;cells\right]}\;\times \;100$$

### 2.8. Gene Expression Studies

Luciferase expression and receptor binding studies were conducted as previously reported [37,38]. Cells were seeded into 48-well plates and incubated as in Section 2.7. Following incubation, the medium was then replaced with 200 µL fresh growth medium. The nanocomplexes were added at the sub-optimum, optimum, and supra-optimum ratios, and cells were incubated at 37 °C for 48 h. The controls included naked pDNA and untreated cells. After the 48 h incubation, the cells were washed twice with 0.2 mL PBS and lysed with 80 µL reporter lysis buffer. The cells were then gently scraped off the wells, and the cell suspension was centrifuged at 12,000× *g* for 30 s using an Eppendorf 5418 centrifuge (Merck, Darmstadt, Germany). After that, 20 µL of each of the cell lysates was added to the respective wells in a white 96-well plate, followed by automatic injection of 100 µL of the luciferase assay reagent. Luminescence was measured using a Glomax^®^ multi-detection system (Promega Biosystems, Sunnyvale, CA, USA). The relative light units obtained were normalized against the protein content measured using the standard Bicinchoninic acid assay (BCA) (Sigma, St. Louis, MO, USA). The readings were expressed as relative light units per milligram of total protein (RLU/mg protein). Each data point was averaged over three replicates (*n* = 3), and the results were presented as means ± SD. A competition assay was performed as above to confirm folate receptor uptake, except for adding a 50 times excess of free folic acid 30 min prior to nanocomplex addition.

### 2.9. Statistical Analysis

Statistical analysis was conducted via GraphPad Prism 9 and OriginLab 2021. Data are presented as a mean ± standard deviation (SD; *n* = 3). Statistical analysis among mean values was performed using a two-way multiple ANOVA. All statistics were performed using a 95% confidence interval (CI) and were considered to be significant at *p* < 0.05.

## 3. Results and Discussion

### 3.1. Characterization of Nanoparticles and Nanocomplexes

#### 3.1.1. UV-Visible Spectroscopy and X-ray Diffraction (XRD)

**Figure 3:**
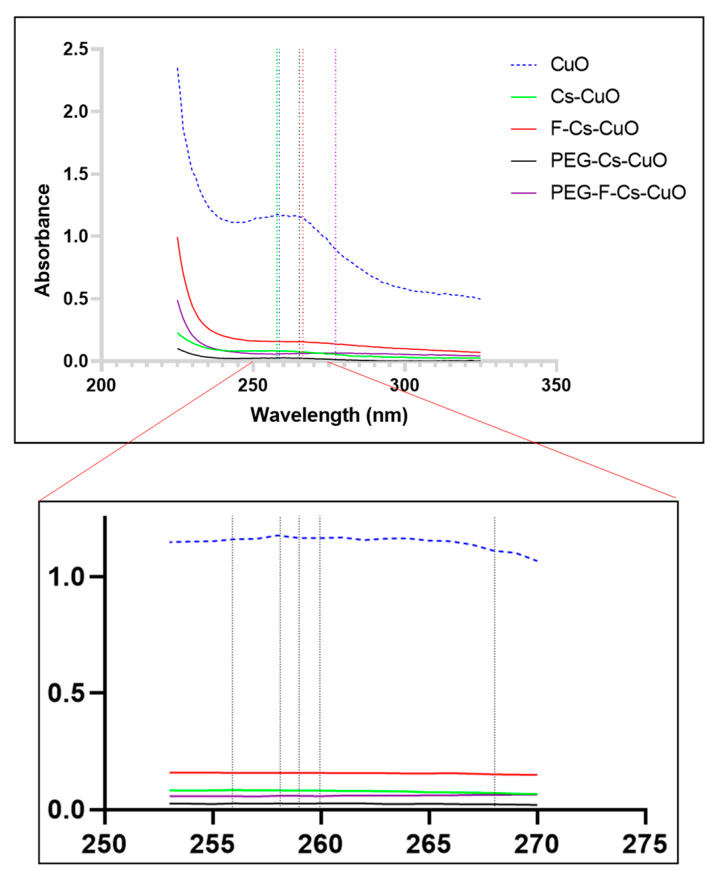
UV-vis spectra of CuONPs, their functionalized counterparts, and an amplified section of the UV-vis spectra showing the slight peak variations among the nanoparticles.

The initial confirmation of the synthesis of the CuONPs was a visual color change from light blue to light yellow/green. This also indicated the excitation of the surface plasmon vibration of the CuONPs, allowing for their spectroscopic measurement. UV-vis spectroscopy (Figure 3) revealed a maximum absorbance peak at 258 nm for the CuONPs.

As observed in the literature, absorbance between 250 and 260 nm was noted and confirmed the synthesis of the CuONPs [39,40]. Upon the conjugation of chitosan, folate, and PEG, a slight shift in the λ_max_ was evidenced. The Cs-CuONPs peaked at 256 nm, while the F-Cs-CuONPs peaked at 266 nm. This shift is due to the successful modifications of the surface of the NPs [40]. Upon PEGylation, PEG-Cs-CuONPs and PEG-F-Cs-CuONPs had an SPR at 260 nm and 276 nm, respectively. Furthermore, the single visible peaks are evidence of a lack of by-products of the leaf extract used in the reduction process. An overall decline in the absorbance was noted due to the dilution of the original solution during the conjugation of chitosan, folate, and PEG.

The peaks seen in the XRD of the CuONPs (Supplementary Figure S1) were similar to those reported previously [41]. There is an agreement with the observed peaks upon the comparison of the data with JCPDS no. 00-048-1548. The strong peaks indicated the high crystalline state of the CuONPs [42].

#### 3.1.2. Fourier-Transform Infrared Spectroscopy (FTIR)

**Figure 4:**
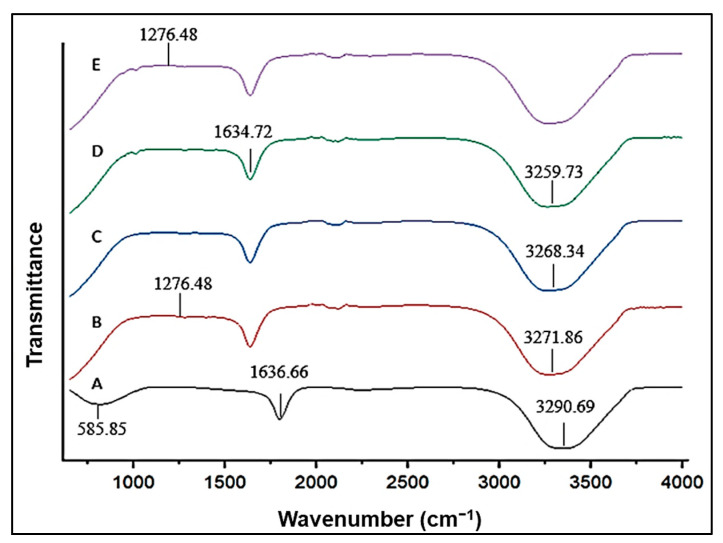
FTIR analysis of (A) CuONPs, (B) Cs-CuONPs, (C) PEG-Cs-CuONPs, (D) F-Cs-CuONPs, and (E) PEG-F-Cs-CuONPs.

Fourier-transform infrared spectroscopy plays an important role in the identification of the functional groups present in a molecule. It also serves as a validation for the synthesis of the CuONPs. Each chemical bond has a specific and unique energy absorption band. Figure 4 portrays the spectrum generated following FTIR analysis. A clear, broad band at 3290.69 cm^−1^ indicated the OH- and C-O groups on the NP’s surface. Stretching vibrations of the Cu-O bonds are characterized by the appearance of a narrower band at 1636.66 cm^−1^. The peak at 585.85 cm^−1^ is ascribed to the formation of the CuONP.

The FTIR spectrum of the Cs-CuONPs (Figure 4) depicts a sharp peak at 1276.48 cm^−1^. This peak indicates complex vibrations of the NHCO group (amide III bands) [43]. The spectrum shows structural changes owing to successful Cs conjugation. The distortion in the peaks at 1913.52 to 2304.16 cm^−1^ is due to the cross-coordination of the Cu ions and the Cs chains [44]. Following conjugation, the broad band at 3700–3100 cm^−1^ shifted to a lower frequency with a more asymmetrical appearance. This is due to the Cu ions complexing with the OH and NH_2_ groups. A further reduction in frequency was noted for PEG-Cs-CuO (Figure 4), owing to the conjugation of PEG.

The subsequent addition of folic acid (Figure 4) produced a wider band at 1634.721 cm^−1^, owing to the carboxylic acid group binding to the Cs-CuONPs. Furthermore, the band at 3259.729 cm^−1^ indicated the -NH and -OH groups of folic acid, correlating with the literature [45]. The peak at 1618 cm^−1^ was due to the conjugation of folic acid to the CuONPs [46]. A further peak at 1278.329 cm^−1^ for the PEG-F-Cs-CuONPs (Figure 4) was noted for the PEG-Cs-CuONPs (Figure 4), confirming the successful coupling of PEG to the NPs.

#### 3.1.3. Transmission Electron Microscopy (TEM)

**Figure 5:**
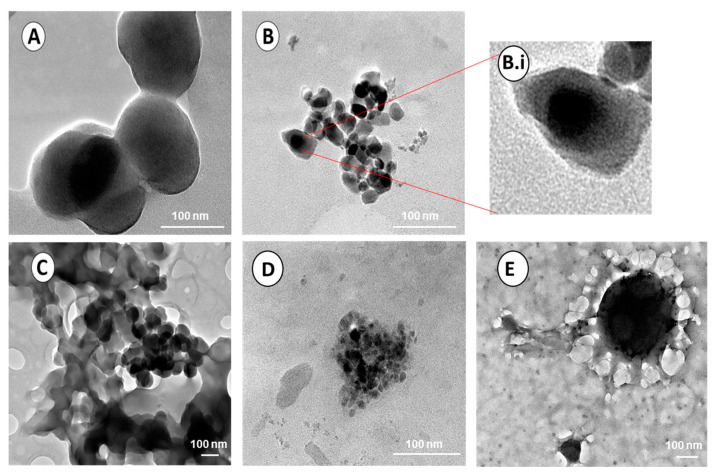
TEM micrographs of the nanoparticles. Bar scale = 100 nm. (**A**) CuONPs, (**B**) Cs-CuONPs, (**B.i**) Cs-CuONPs (zoomed), (**C**) F-Cs-CuONPs, (**D**) PEG-Cs-CuONPs, and (**E**) PEG-F-Cs-CuONPs.

TEM provides a means of analyzing the ultrastructural morphology of the NPs together with their size and dispersity. Figure 5A shows that the CuONPs appear as smooth, spherical particles ranging from 48 nm to 69.5 nm, with an average size of 62.8 ± 12.8 nm (Table 1). Figure 5B depicts the Cs-CuONPs as clusters, maintaining their initial shape, with a larger size of 85.7 ± 26.9 nm (size range from 68.6 nm to 116.6 nm). The F-Cs-CuONPs (Figure 5C) demonstrated sizes ranging from 105.2 nm to 127.8 nm with an average size of 114.4 ± 11.9. nmTEM showed that the PEGylated NPs were larger, following the trend seen for the NTA (Section 3.1.4). In addition, the molecular weight of the PEG used may be a good indicator of the extent to which the NPs’ size will increase, with a higher molecular weight possibly leading to larger NPs. The present study utilized PEG2000, which produced a significant size difference between the PEGylated and unPEGylated nanocomplexes. The PEG-Cs-CuONPs ranged from 87.3 nm to 98.2 nm, while the F-Cs-CuONPs were 125.5 nm to 159.5 nm. However, no significant difference was noted in the shape of the F-Cs-CuONPs, upon PEG addition, aside from the size increase.

A thin coating around the NPs due to Cs coating was also observed (Figure 5B.i). The small size difference noted with that of CuONPs and Cs-CuONPs is in line with the slight red shift noted for UV-vis spectroscopy. The NPs showed minimal aggregation, which is not uncommon, and required sonication before treating the cells. This aggregation did not affect the stability of the NPs and their nanocomplexes, as evidenced by the zeta potentials obtained from the NTA analysis (Section 3.1.4).

#### 3.1.4. Nanoparticle Tracking Analysis (NTA)

The biocompatibility of NPs depends on their size, shape, and charge. NTA combines laser light scattering microscopy and a charged-coupled device (CCD) camera to enable an accurate visualization of the NPs [47]. The analysis allows for the individual NPs to be tracked based on their Brownian motion in real-time, making NTA a very sensitive and robust analytical system. NTA accurately measured the size and zeta(ζ) potential of the NPs, as reported in Table 2. Although differences can be observed between the particle sizes obtained from the NTA and TEM, the general trend in the size increase was noted for the functionalized NPs. This difference in size may be attributed to the different sample preparation methods, with TEM using samples that were dried on a copper grid, while NTA utilized samples in aqueous solutions and measured the samples in real-time [31].

Unmodified CuONPs exhibited the smallest size and a low negative ζ-potential (78.2 ± 20.7 nm; −9 ±0.1 mV). This negative charge was consistent with studies conducted by Sarkar et al. (2020) who observed a ζ potential of −2.67 mV. It has been proposed that the NPs’ slight negative charge may be beneficial to prevent aggregation [48,49]. Upon modification with Cs, a notable increase in size and ζ-potential was seen (103.9 ± 14.8 nm; 45.3 ± 0.1 mV), which confirmed the successful chitosan conjugation. Furthermore, the positive charge indicates the presence of the cationic amino groups of Cs on the CuONPs. These results correlate to those obtained via UV-vis spectroscopy.

The conjugation of folate resulted in a further increase in the size and ζ-potential (128.0 ± 9.4 nm; 55.1 mV ± 0.1), confirming the successful binding of the folate to the NP. The positive charge is advantageous as it can support electrostatic interactions with the pDNA and anionic cellular membrane. The NPs portrayed a general trend in the increase in size and ζ-potential (mV) upon adding the polymer and ligand. This further confirmed that the Cs effectively stabilized the CuONPs and prevented aggregation.

The subsequent PEGylation of the Cs-CuONPs produced a slight decrease in ζ-potential. This decline may be due to the PEG chains masking the amino groups on the Cs. Furthermore, it has been reported that the molecular weight and density of PEG can increase the thickness of the PEG layer, resulting in a lowered ζ-potential, which could reach a neutral state [50]. Nanocomplexes containing PEG2000 have also been reported to be larger than those containing a lower-molecular-weight PEG [33]. However, the PEG-Cs-CuONPs and the PEG-F-Cs-CuONPs showed a further increase in the NP size, with improved ζ-potential. This increase in size further served as confirmation of successful PEG conjugation. Early research studies reported that nanomaterials functionalized with folate could utilize clathrin- and caveolae-independent endocytosis pathways for cellular uptake [51].

NPs that have ζ-potential values higher than +30 mV or lower than −30 mV are proposed to have excellent colloidal stability [31], as seen for the functionalized NPs and some of the nanocomplexes. The improved ζ-potential upon folate binding could have resulted in an altered PEG conformation on the NP, allowing for the availability of more of the cationic charges of the Cs. The changes in ζ-potential of the nanocomplexes were expected and confirmed the successful binding of the anionic DNA to the cationic NP. NPs with ζ-potentials not within the favorable range may experience some aggregation, since the attractive forces may exceed or equal the repulsive forces between the NPs. Although the ζ-potential for the PEG-Cs-CuO nanocomplex was reduced (19.7 mV), values around 20 mV have been reported to provide adequate stability [52]. However, this ζ-potential value proved to be sufficient to offer stability to the nanocomplex and facilitate favorable cellular uptake and transfection.

### 3.2. DNA Binding Studies

#### 3.2.1. Electrophoretic Mobility Shift Assay

**Figure 6:**
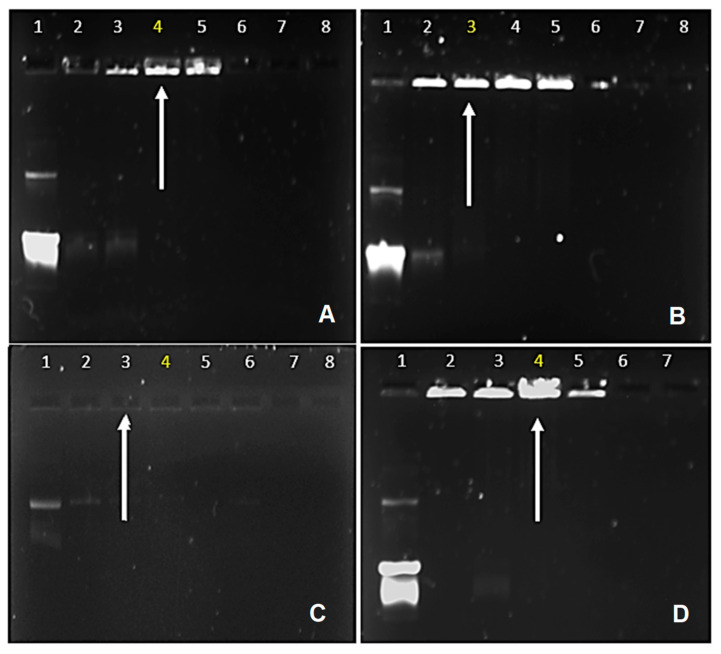
Electrophoretic mobility shift assay of the nanocomplexes. Lane 1 represents the positive control, consisting of naked pDNA (0.25 µg/mL). (**A**) Cs-CuONPs, lanes 1–5 (0, 0.2, 0.4, 0.6, 0.8 µg), (**B**) F-Cs-CuONPs, lanes 1–5 (0, 0.2, 0.4, 0.6, 0.8 µg), (**C**) PEG-Cs-CuONPs, lanes 1–5 (0, 0.1, 0.2, 0.3, 0.4, 0.5 µg), and (**D**) PEG-F-Cs-CuONPs, lanes 1–5 (0, 0.1, 0.2, 0.3, 0.4 µg). White arrows/yellow numbers indicate optimum binding ratios, preceded by the sub-optimum ratio and followed by the supra-optimum ratio.

The principle of this assay works based on the cationic polymeric NPs electrostatically interacting with the negatively charged pDNA, forming nanocomplexes of varying charge ratios. Upon electroneutrality, the mobility of the pDNA into the gel is hindered, allowing for the determination of the point of complete condensation of the pDNA by the functionalized CuONPs (Figure 6). The white arrows in Figure 6 indicate the points of electroneutrality and the optimum binding (*w*/*w*) ratio. It can be seen that the F-Cs-CuONPs bind pDNA at a lower ratio (*w*/*w*) (0.4:1) compared to the Cs-CuONPs (0.6:1). A binding ratio of 0.3:1 (*w*/*w*) was obtained for both the PEG-Cs-CuONPs and PEG-F-Cs-CuONPs. There was some correlation with the NTA results, which showed an increase in the positive ζ-potential upon the conjugation of Cs, F, and PEG to the NPs.

The increased charge enhanced the complexation of the pDNA. The assay confirmed that these NPs have excellent pDNA binding capability. The inability of the DNA to migrate out of the wells at low ratios is in accordance with an early study [53] that showed the high compaction ability of chitosan. In some instances, such as in Figure 6C, the nanocomplexes formed are electroneutral and tend to float out of the wells. Hence, they are not seen as fluorescent bands in the wells. Similar results were seen before, such as in [37]. The low ratio of the F-Cs-CuONPs required in the binding of the pDNA further suggests that the covalently linked folate did not affect the DNA compaction ability of the chitosan.

#### 3.2.2. Nuclease Protection Assay

**Figure 7:**
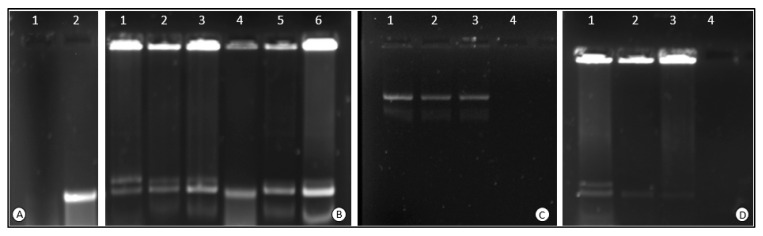
The serum nuclease protection assay. (**A**) Lane 1 contains the negative control (pDNA treated with FBS), while lane 2 contains the positive control of only naked pDNA (0.25 µg). (**B**) Lane 1–3: Cs-CuONPs (0.4, 0.6, 0.8 µg); lanes 4–6: F-Cs-CuONPs (0.2, 0.4, 0.6 µg). (**C**) Lanes 1–3: PEG-Cs-CuONPs (0.2, 0.3, 0.4 µg). (**D**) Lanes 1–3: PEG-F-Cs-CuONPs (0.2, 0.3, 0.4 µg). All nanocomplexes were complexed to pDNA (0.25 µg) and treated with 10% FBS.

A major intracellular challenge to a gene delivery system is the presence of various degradative enzymes, such as nucleases, resulting in the premature clearance of the genetic material in the bloodstream [54]. The carrier must protect the cargo during transportation for successful therapy to occur. The present assay assesses the ability of the NPs to protect the pDNA upon entering a simulated in vivo environment. Successful protection is visualized as distinct pDNA bands on the gel. This can be seen for all of the nanocomplexes (Figure 7). These bands confirm that the NPs can effectively protect the bound pDNA. The negative control (Figure 7A, lane 1) showed complete degradation of the naked pDNA.

In most cases, much of the DNA remained NP-bound in the wells, suggesting that the SDS used was insufficient to release the DNA. Alternate chemicals or increased SDS concentrations may be needed to obtain the complete release of the DNA. Nevertheless, this still confirmed the protection ability of the NPs. Similar results showing an incomplete release of the pDNA by SDS were noticed for metallic NPs [55].

#### 3.2.3. Ethidium Bromide Intercalation Assay

**Figure 8:**
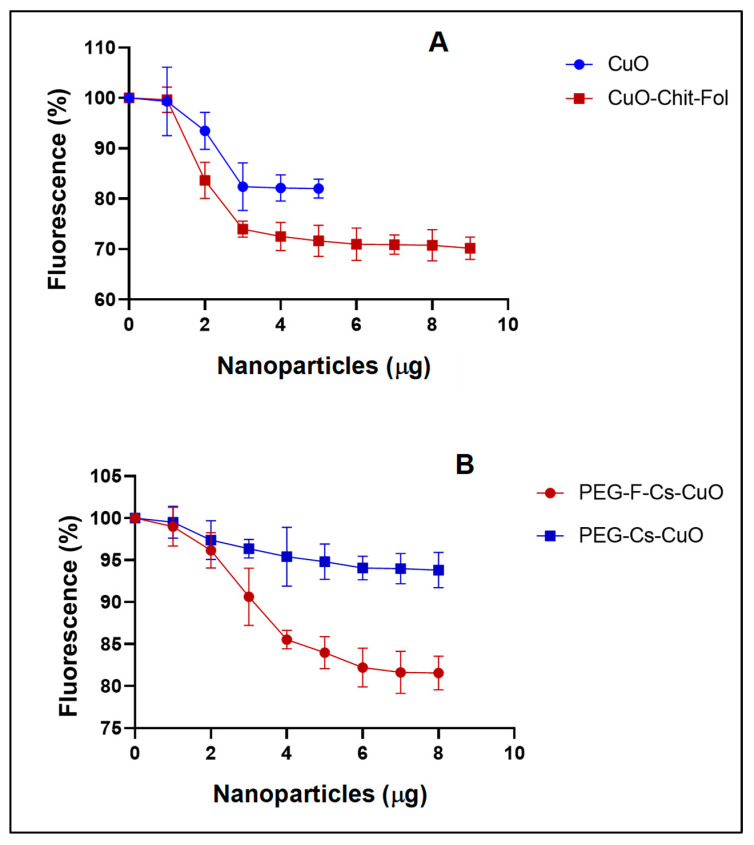
Ethidium bromide intercalation assay of the nanocomplexes. Incubation mixtures contained pCMV-*Luc* DNA (2.5 µg) in 100 µL HBS, with the addition of increasing amounts of the (**A**) Cs-CuONPs and F-Cs-CuONPs, and (**B**) PEG-Cs-CuONPs and PEG-F-Cs-CuONPs.

Ethidium bromide is a cationic fluorescent dye that can intercalate between the DNA bases. The fluorescence is measured while increasing the concentration of cationic NPs, to determine the degree of compaction of the pDNA by the NPs. The complete complexation of the nanocomplex is indicated by a plateau in fluorescence readings. Figure 8A,B showed a steady decrease in fluorescence, correlating with an increase in NP concentration. The F-Cs-CuONPs produced a greater pDNA compaction of 82.4% compared to that of the Cs-CuONPs (71%).

Furthermore, the PEGylated nanocomplex showed increased compaction, as seen for PEG-Cs-CuONPs (84%) and PEG-F-Cs-CuONPs (95.4%). These results followed a similar trend to the electrophoretic mobility shift assay. The nanocomplexes must be compact enough to protect the nucleic acid cargo but not too tight to prevent disassociation of the nucleic acid from the nanoparticle. Overall, the binding of the NPs to the pDNA was sufficient to facilitate efficient gene expression, as observed in this study.

### 3.3. MTT Cell Viability Assay

The efficacy of gene therapy is dependent on its ability to target cancer cells while ensuring no adverse effects on healthy cells. The MTT assay was employed to determine the cell viability, measured in terms of the loss of metabolism in dead cells and the inability of the mitochondrial dehydrogenase enzyme to reduce MTT to formazan [37]. The cell viabilities (%) were expressed relative to the control of untreated cells (100%). The sub-optimum, optimum, and supra-optimum ratios obtained from the electrophoretic mobility shift assay (Section 3.2.1) were utilized. This was conducted to minimize the risk of any unconjugated CuONPs entering the cells and enabled the comparison between the three binding ratios, in order to ascertain which ratio produced the best transgene expression.

**Figure 9:**
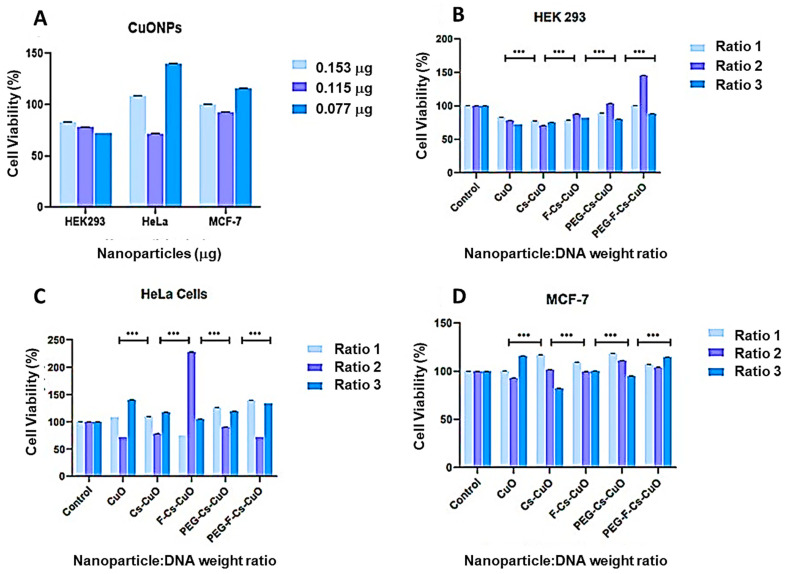
MTT assay portraying the cell viability of (**A**) CuONPs (0.153 µg; 0.115 µg; 0.077 µg), together with the nanoparticles in (**B**) HEK293, (**C**) HeLa, and (**D**) MCF-7 cells. The ratios are as follows: Cs-CuO (0.4:1; 0.6:1; 0.8:1), F-Cs-CuO (0.2:1; 0.4:1; 0.6:1), PEG-Cs-CuO (0.3:1; 0.4:1; 0.5:1), and PEG-F-Cs-CuO (0.2:1; 0.3:1; 0.4:1). Data are represented as means ± SD (*n* = 3). *** *p* < 0.001. was considered to be statistically significant.

The promotion of cellular growth by the CuONPs was noted in the HeLa and MCF-7 cells, with a dose-dependent effect evident for the HEK293 cell (Figure 9A) with cell viability exceeding 70%. This is in keeping with Cus’ natural properties of good biocompatibility and biodegradability [8]. It was proposed that an increased concentration of CuONPs would result in more significant cellular accumulation, resulting in enhanced stress and cell death [56]. A study by Fard et al. (2020) showed that CuONPs exhibited a dose-dependent cytotoxicity on peripheral blood cells, with a low concentration of CuONPs not significantly affecting the cell viability [57]. Overall, the nanocomplexes were well tolerated in all cells tested (HEK293, HeLa, and MCF-7), with cell viability >70% (Figure 9B–D). The increased cell viability may be due to the bound chitosan in the nanocomplexes, which can interact with the cell membranes and stimulate growth factors [58,59]. In addition, chitosan has been employed in tissue engineering [60], highlighting its suitability as a functionalizing polymer.

The targeted F-Cs-CuONPs portrayed a significantly high cell viability, especially at the optimum binding ratio in the HeLa cells, which was greater than that of the control. Similar high cell viabilities were noted in the HEK293 and MCF-7 cells. A study by Mansouri et al. (2006) revealed 79% cell viability in HEK293 cells using folate-targeted chitosan NPs. This affirms that folate does not affect the properties exhibited by chitosan but rather enhances them [53]. The additional conjugation of PEG to the functionalized NPs (Cs-CuO and F-Cs-CuO) showed a further increase in viability in the HEK293 cells, and a moderate change in the viabilities of the HeLa and MCF-7 cells, compared to the unPEGylated nanocomplexes. A recent study reported that adding PEG to their NPs (Fe_3_O_4_) improved the NP stability and biocompatibility in 3T3 and HepG2 cells, preventing any toxic effects of the NPs due to their interactions with the cells or proteins [61]. Contrary to this, an earlier study using MgFe_3_O_4_ NPs noted that adding PEG increased cytotoxicity in breast cancer cells (SKBR-3). The authors attributed this to the possible interference of the function and structure of the cell membranes by the PEG molecules [62,63]. This study’s overall low cytotoxicity is desirable, as it confirms the nanocomplexes’ ability to be safe for gene delivery, especially regarding cervical cancer cells.

### 3.4. Gene Expression Studies

**Figure 10:**
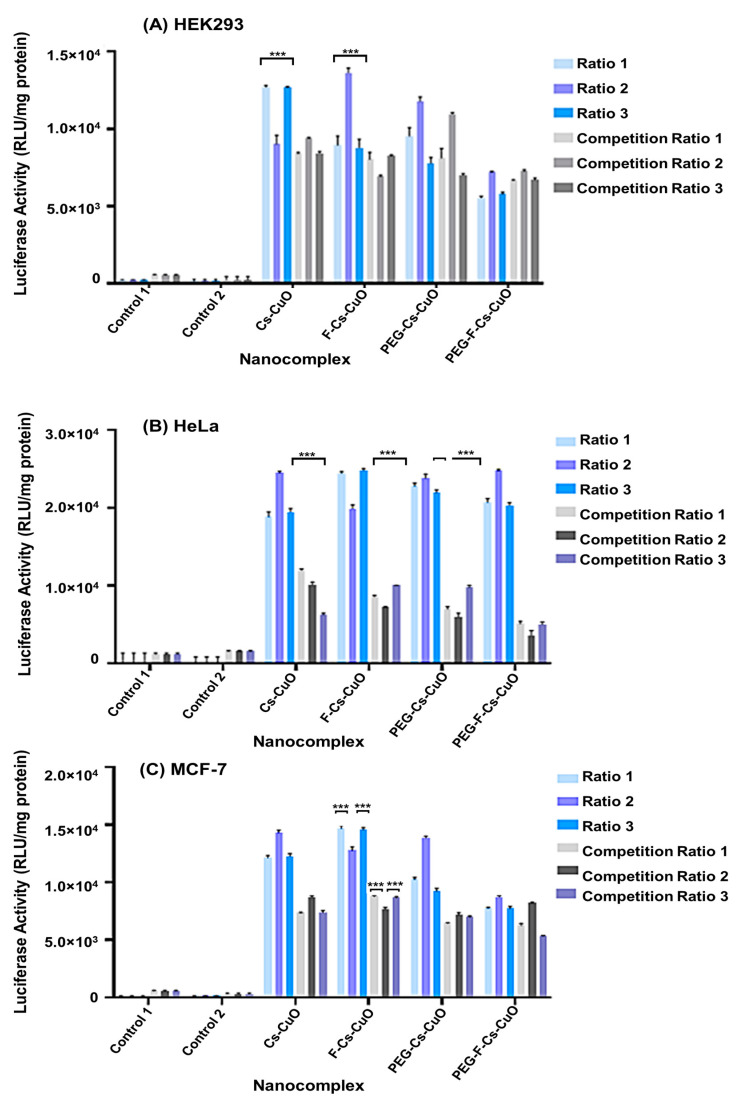
In vitro luciferase activity and competition studies in (**A**) HEK293, (**B**) HeLa, and (**C**) MCF-7 cells. Each column represents the mean ± SD (*n* = 3). Luciferase expression was measured in RLU/mg protein. *** *p* < 0.001 was considered to be statistically significant. The ratios were represented as follows: Cs-CuO (0.4:1; 0.6:1; 0.8:1), F-Cs-CuO (0.2:1; 0.4:1; 0.6:1), PEG-Cs-CuO (0.2:1; 0.3:1, 0.4:1), and PEG-F-Cs-CuO (0.2:1, 0.3:1, 0.4:1).

This assay confirmed the potential of these Cu-based nanocomplexes in gene delivery and their ability to facilitate folate-receptor-mediated endocytosis (Figure 10). HEK293 cells served as control cells that were non-cancerous and lacking folate receptors. In the HEK293 cells, higher transgene expression was noted for the Cs-CuONPs and F-Cs-CuONPs compared to their PEGylated counterparts. This can be attributed to PEG shielding some of the cationic charges of Cs and the larger sizes of these PEG nanocomplexes. A key factor in determining transfection efficiency has been proposed to be the size of NPs [64]. NPs in the 120 to 150 nm range exhibit cellular internalization via clathrin- or caveolin-mediated endocytosis [65,66], with the optimum size limits being 50–100 nm [67]. NPs above (70–240 nm) and below (15–30 nm) have been reported to exhibit a decline in cellular uptake and transfection ability [68,69]. This is in keeping with the favorable transfection ability revealed by the F-Cs-CuONPs and Cs-CuONPs. This is further depicted in the difference between the transfection efficacy of PEG-Cs-CuONPs (91.8 ± 9.4 nm) and PEG-F-Cs-CuONPs (139.3 ± 17.9 nm), with the former showing greater transgene expression. A study using silver NPs noted that smaller particles might accumulate and contribute to lung damage compared to larger NPs [70].

The HeLa cells exhibited the highest transgene expression, followed by the MCF-7 and HEK293 cells. All nanocomplexes did perform almost equally well in the HeLa cells, suggesting that uptake occurred via various processes, including clathrin- or caveolae-mediated endocytosis and receptor-mediated endocytosis for the targeted nanocomplexes. Upon further examination using the competition assay, the PEG-F-Cs-CuO nanocomplexes showed cellular uptake via folate receptor mediation, as evidenced by the significantly lowered transgene expression obtained after the addition of excess folate which bound to and blocked the folate receptors overexpressed on the HeLa cells, preventing the entry of the PEG-F-Cs-CuO nanocomplexes. The size of the nanocomplexes was not the main determining factor in cellular internalization in this cell line, as the nanocomplex sizes varied. It was reported that different cell lines possessed different properties pertaining to their cell surfaces, resulting in a varied cellular uptake mechanism due to the range of NP sizes [71]. This study noted that uptake in the HeLa cells increased with an increase in NP size, while in the MCF-7 cells, a reduced uptake occurred with larger NPs. This was confirmed by the lack of significant reduction in the transgene expression in the PEGylated nanocomplexes compared to the unPEGylated nanocomplexes, as reported in previous studies [72,73].

The MCF-7 cells had fewer folate receptors on their cell surface than the HeLa cells, which was borne out via the significant differences in the transfection activities of the targeted nanocomplexes (PEG-F-Cs-CuO and F-Cs-CuO). Marshalek et al. (2016) compared the breast cancer cells MCF-7 and MDA-MB-231, and showed that the MCF-7 cells portrayed 1.76 times fewer folate receptors [74]. Another study examined the difference in folate receptors in the MCF-7 and HeLa cells utilizing nanoprobes, which revealed strong fluorescence in the HeLa cells, with very little fluorescence observed in the MCF-7 cells, validating the results obtained in the present study [75].

Overall, the transfection activity of all of the nanocomplexes declined in all of the cells in the competition assay. This could be due to the excess folate either blocking the receptors and preventing receptor-mediated endocytosis of the targeted nanocomplexes or their presence on the cell surface posing a challenge for the untargeted nanocomplexes to enter the cells via passive endocytosis, maropinocytosis, or micropinocytosis. Nevertheless, this study did confirm the uptake of the targeted nanocomplexes, especially the PEG-F-Cs-CuO nanocomplexes, via folate-receptor-mediated endocytosis. The addition of PEG did seem to improve the targeted delivery of these nanocomplexes. However, there is a need for further studies on the cellular uptake mechanisms involved and a need to improve the current system for enhanced targeted delivery.

## 4. Conclusions

Holistically, this study demonstrated that the formulated CuONP-based nanocomplexes could safely and efficiently deliver the pDNA to cervical cancer cells in vitro. Our findings highlight the potential of folate-targeting for cancer therapy, and the benefits of the carrier systems’ physiochemical properties for pharmaceutical research and future clinical applications. Hence, further investigations and optimizations are warranted to improve efficiency and to assess their in vivo applicability. Future studies could include optimizing the green synthesis approach by utilizing other biological materials, testing the nanocomplexes in a co-culture environment, i.e., with normal and cancer cells synergistically, and optimizing the receptor-mediated uptake in cervical cancer cells for enhanced transgene activity. Mechanistic studies to understand the cellular uptake and trafficking of the nanocomplexes in the cells would be beneficial. Overall, our study provides a robust tool for gene delivery studies and underscores the potential of gene therapy for cancer treatment. This study vaunts a non-invasive approach, laying the foundation for using other biological materials in NP synthesis. While CuONPs have been synthesized before via chemical, physical, and biological means, the novelty of this study lies in the gene delivery potential of the nanoconjugates produced, and the *M. azedarach* leaf extracts utilized, which acted synergistically with the NPs to achieve the desired therapeutic effect.

## Figures and Tables

**Table 1:** Particle size (nm) obtained from TEM imaging (*n* = 3).

Nanoparticles	Particle Size (nm) ± SE
CuO	62.8 ± 12.8
Cs-CuO	85.7 ± 26.9
F-Cs-CuO	114.4 ± 11.9
PEG-Cs-CuO	91.8 ± 9.4
PEG-F-Cs-CuO	119.3 ± 17.9

**Table 2:** Particle size (nm) and zeta potential (mV) of the nanoparticles.

Sample	Particle Size (nm) ± SE		ζ Potential (mV) ± SE	
	Nanoparticle	Nanocomplex (NP + pDNA)	Nanoparticle	Nanocomplex (NP + pDNA)
CuO	78.2 ± 20.7	-	−9 mV ± 0.1	-
Cs-CuO	103.9 ± 14.8	159.3 ± 6.7	45.3 mV ± 0.1	24.2 mV ± 0.1
F-Cs-CuO	128.0 ± 9.4	161.5 ± 9.5	55.1 mV ± 0.1	30.3 mV ± 0.2
PEG-Cs-CuO	148.8 ± 2.3	178.3 ± 3.7	42.3 mV ± 0.2	19.7 mV ± 0.1
PEG-F-Cs-CuO	197.1 ± 7.9	209.0 ± 9.8	55.5 mV ± 0.1	35.1 mV ± 0.1

## Data Availability

All data presented in the study are included in the article. Further inquiries can be directed to the corresponding author.

## Supplementary Materials

The following supporting information can be downloaded at https://www.mdpi.com/article/10.3390/polym15102393/s1: Figure S1: XRD of copper oxide nanoparticles.
