# Supplementary material for: Copolymer-Green-Synthesized Copper Oxide Nanoparticles Enhance Folate-Targeting in Cervical Cancer Cells In Vitro
Source: Polymers (Basel). 2023 May 20;15(10):2393. doi: 10.3390/polym15102393 (PMC10223539; doi:10.3390/polym15102393)
Supplement: Supplementary file 1 [file polymers-15-02393-s001.zip › polymers-2346652-supplementary.pdf]

polymers

Supplementary

Copolymer-Green-synthesized Copper Oxide Nanoparticles enhance folate-targeting in cervical cancer cells in vitro

Keelan Jagaran [1] and Moganavelli Singh [1,*]

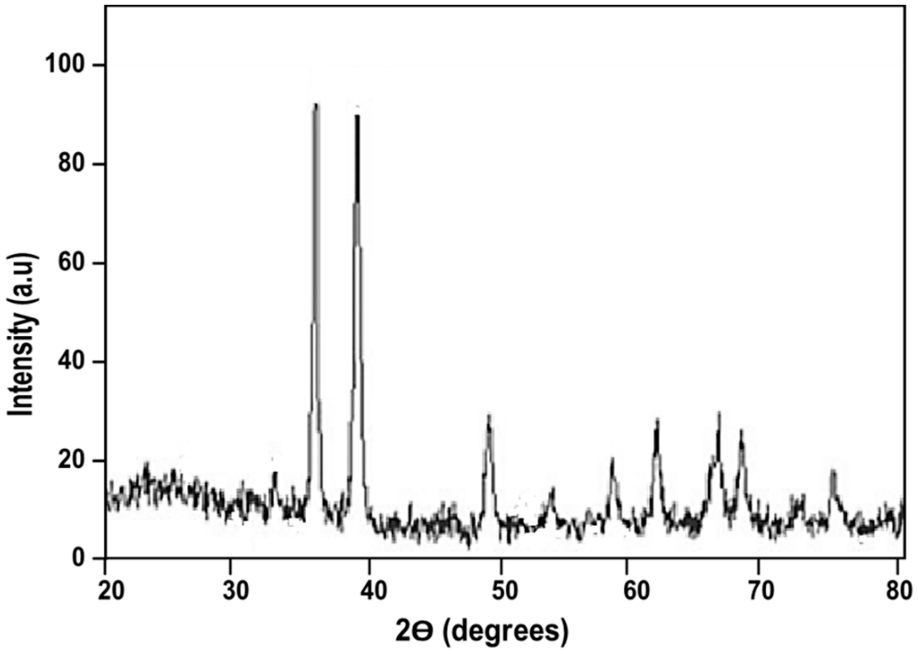

Supplementary Figure S1: XRD of copper oxide nanoparticles.
